# Functional near-infrared spectroscopy-based correlates of prefrontal cortical dynamics during a cognitive-motor executive adaptation task

**DOI:** 10.3389/fnhum.2013.00277

**Published:** 2013-07-04

**Authors:** Rodolphe J. Gentili, Patricia A. Shewokis, Hasan Ayaz, José L. Contreras-Vidal

**Affiliations:** ^1^Department of Kinesiology, School of Public Health, University of MarylandCollege Park, MD, USA; ^2^Graduate Program in Neuroscience and Cognitive Science, University of MarylandCollege Park, MD, USA; ^3^Maryland Robotics Center, University of MarylandCollege Park, MD, USA; ^4^Cognitive Neuroengineering Quantitative Experimental Research Collaborative, Drexel UniversityPhiladelphia, PA, USA; ^5^School of Biomedical Engineering, Science, and Health Systems, Drexel UniversityPhiladelphia, PA, USA; ^6^Nutrition Sciences Department, College of Nursing and Health Professions, Drexel UniversityPhiladelphia, PA, USA; ^7^Laboratory for Non-invasive Brain-Machine Interface Systems, Department of Electrical and Computer Engineering, University of HoustonHouston, TX, USA

**Keywords:** visuomotor adaptation-learning, frontal executive, functional near infrared spectroscopy, internal models, arm reaching movement

## Abstract

This study investigated changes in brain hemodynamics, as measured by functional near infrared spectroscopy, during performance of a cognitive-motor adaptation task. The adaptation task involved the learning of a novel visuomotor transformation (a 60° counterclockwise screen-cursor rotation), which required inhibition of a prepotent visuomotor response. A control group experienced a familiar transformation and thus, did not face any executive challenge. Analysis of the experimental group hemodynamic responses revealed that the performance enhancement was associated with a monotonic reduction in the oxygenation level in the prefrontal cortex. This finding confirms and extends functional magnetic resonance imaging and electroencephalography studies of visuomotor adaptation and learning. The changes in prefrontal brain activation suggest an initial recruitment of frontal executive functioning to inhibit prepotent visuomotor mappings followed by a progressive de-recruitment of the same prefrontal regions. The prefrontal hemodynamic changes observed in the experimental group translated into enhanced motor performance revealed by a reduction in movement time, movement extent, root mean square error and the directional error. These kinematic adaptations are consistent with the acquisition of an internal model of the novel visuomotor transformation. No comparable change was observed in the control group for either the hemodynamics or for the kinematics. This study (1) extends our understanding of the frontal executive processes from the cognitive to the cognitive-motor domain and (2) suggests that optical brain imaging can be employed to provide hemodynamic based-biomarkers to assess and monitor the level of adaptive cognitive-motor performance.

## Introduction

Humans have the ability to adapt their movements to various environments and/or perturbations through practice or experience. A possible approach to investigate human adaptation capabilities is to simultaneously examine the brain dynamics and behavioral changes during arm movements in the presence of a visual distortion of the movement trajectory (e.g., Contreras-Vidal and Kerick, 2004; Anguera et al., 2007; Seidler and Noll, 2008; Gentili et al., 2009, 2011). Under such conditions, individuals are required to learn the internal representation of the novel visuomotor transformation (i.e., a hand-screen cursor rotation) to perform accurate movements (e.g., Kluzik et al., 2008; Kagerer and Contreras-Vidal, 2009; Gentili et al., 2011). Visuomotor adaptation paradigms require inhibiting prepotent visuomotor mappings that are no longer task-relevant and consequently may interfere with the ongoing adaptation process.

Brain dynamics during visuomotor task adaptations have been investigated by employing various neuroimaging techniques. For instance, numerous studies combined an adaptation task with functional Magnetic Resonance Imaging (fMRI) (e.g., Seidler et al., 2006; Seidler and Noll, 2008). However, the constrained movement amplitudes and the unnatural placement of the subject's body in a supine position while performing the task in a magnet provided limited ecological validity; since daily physical motor activities are usually performed in a seated or standing position. To address the issues of limited ecological validity and task performance in natural settings, functional near infrared spectroscopy (fNIR) enables monitoring of cortical activity in natural settings was used (e.g., Hatakenaka et al., 2007; Ikegami and Taga, 2008; Leff et al., 2008a,b; Ayaz et al., 2009, 2011, 2012a,b,c; Ohuchida et al., 2009; James et al., 2010, 2012; Gentili et al., 2010a). In addition, there are few magnetoencephalography (MEG) and electroencephalography (EEG) investigations of brain dynamics during performance of visuomotor adaptation tasks (Contreras-Vidal and Kerick, 2004; Anguera et al., 2009; Bradberry et al., 2009; Gentili et al., 2009, 2011; Perfetti et al., 2011). These recent EEG studies evidenced a refinement of the cortical dynamics throughout adaptation for individuals facing the distortion whereas no changes in brain dynamics or behavior were observed in individuals who did not face the distortion challenge. However, for individuals who faced the distortion, there was an increase in alpha power in the prefrontal regions that reflect a progressive derecruitment of the prefrontal inhibitory functions. Thus, these prefrontal inhibitory functions are highly engaged during early learning to inhibit the prepotent motor responses whereas they become irrelevant to the task demand during late adaptation (Gentili et al., 2010a, 2011).

Beyond these studies, there is a critical need to investigate hemodynamic changes in ecological situations (e.g., seated positions) as the brain adapts by considering alternative neuroimaging approaches such as fNIR. While EEG provides a measure of neural electrical activity, by contrast fNIR measures blood oxygenation levels via infrared light (e.g., Izzetoglu et al., 2007; Ayaz et al., 2009, 2011, 2012a,b,c). In essence, fNIR can provide different and complementary biological markers for brain dynamics with increased robustness to artifacts during cognitive and motor performance under everyday conditions and in real life environments (e.g., Coyle et al., 2007; Hatakenaka et al., 2007; Leff et al., 2008a,b; Abdelnour and Huppert, 2009; Ayaz et al., 2009, 2011, 2012a,b,c; Gentili et al., 2010a; James et al., 2010, 2012; Power et al., 2012; Sweeney et al., 2012). Comparatively, it was demonstrated that fNIR could indicate various levels of cognitive workload (Izzetoglu et al., 2005; Ayaz et al., 2009, 2012a,b,c; James et al., 2012; Power et al., 2012) as well as changes in motor performance (Hatakenaka et al., 2007; Ikegami and Taga, 2008; Leff et al., 2008a,b; Morihiro et al., 2009; Gentili et al., 2010a).

Among the rare fNIR investigations that focused on motor learning (e.g., Leff et al., 2011; Ayaz et al., 2012a,b,c; James et al., 2012), none investigated adaptive brain dynamics capabilities along with the concomitant changes in performance during a visuomotor adaptation task where individuals faced a cognitive-motor challenge such as the inhibition of prepotent motor responses that are no longer task-relevant.

Therefore, the present study examined functional brain activation by employing fNIR with a particular emphasis on the prefrontal regions since the visuomotor task we employed solicited these specific cortical regions that inhibit prepotent motor responses to facilitate adaptation processes. We predicted that as adaptation happened, there would be a progressive reduction of the cortical activity (i.e., a reduction of oxygenation level) for individuals who experienced the visual distortion since during early adaptation, frontal executive (inhibitory, updating) functions are necessary to adapt to the task demands whereas these same executive functions would become much less relevant by late adaptation. In addition, no cortical or behavioral changes were expected to be observed for individuals who were not exposed to the visual distortions since the engagement of these executive functions was unnecessary.

## Materials and methods

### Participants and apparatus

**Figure 1:**
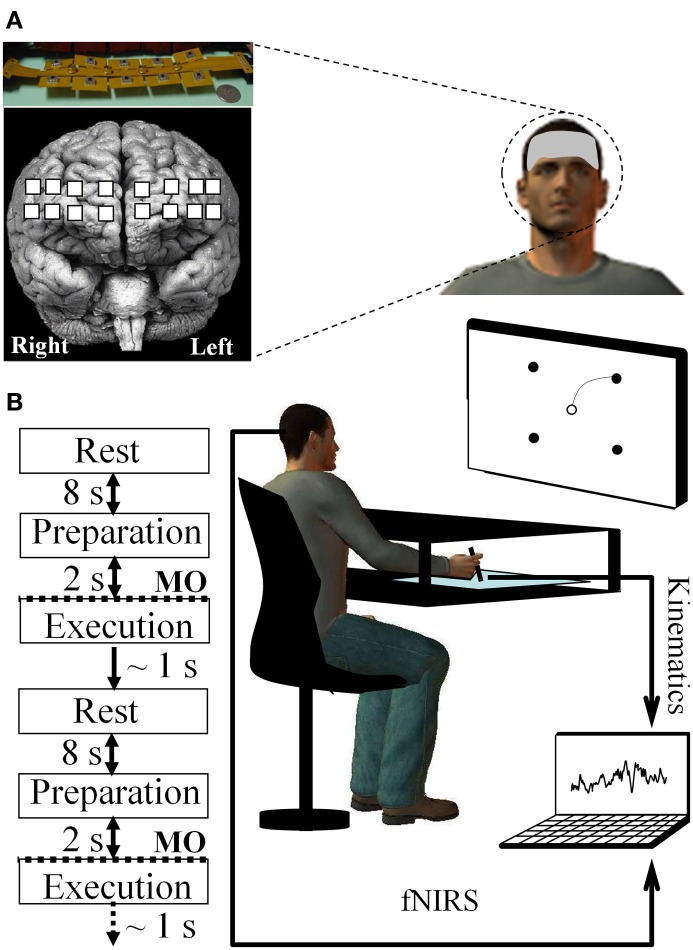
**Experimental setup and devices. (A)** fNIR probe (Drexel University™) including the 16 channels (represented by a filled white square) and its placement on the prefrontal region of the forehead subjects (Ayaz et al., 2012a). **(B)** Sequence composing a reaching trial and set-up allowing for continuously recording of both fNIR and kinematic signals via the digitizing tablet during arm reaching movement. MO, Movement onset.

Twenty-six right-handed and healthy adults (12 males and 14 females ranged from 20 to 35 years old) with normal or corrected-to-normal vision volunteered to participate in this study that was approved by the Institutional Review Board at the University of Maryland-College Park. Participants were seated at a table while facing a computer screen that was placed in front of them at a distance of ~60 cm; while they were required to draw a line by moving a pen on a digitizing tablet (12 WACOM, InTuos). Pen trajectories were displayed in real time as solid black lines on the computer screen by means of custom software (Oasis v.8.29 Kikosoft, Neijmegen); however, a horizontal board prevented vision of the arm/hand moving on the digitizing tablet. Participants had to execute with their right arm/hand “center-out” movements to draw lines from a home target circle (ø = 5 mm) placed in the center of the screen to one of four peripheral target circles (ø = 5 mm). As such, the home target circle represented the origin of a polar frame of reference in which the pointing target circles were positioned at 10 cm from the origin and located at 45°, 135°, 225°, and 315°, respectively (Figure 1). Concurrently, optical brain imaging signals were recorded by the continuous-wave dual-wavelength fNIR system first described by Chance et al. (1993) and developed at Drexel University (Ayaz et al., 2011, 2012a). Accurate and repeatable positioning of the sensor pad was ensured by using the International 10–20 system for electrode placement and by matching the center of the sensor with the vertical axis of symmetry that passes through the nasion. This fNIR system included three components: (1) a flexible headpiece (sensor pad) which incorporates both light sources and detectors enabling therefore a fast placement of all 16 optodes (channels), (2) a control box for hardware processing, and (3) a computer for data acquisition with triggers to synchronize events with the fNIR signal. The sensor had a temporal resolution of 500 milliseconds per scan with 2.5 cm source-detector separation allowing for ~1.25 cm penetration depth. There are four light emitting diodes (LED) that can shine in 730 and 850 nm wavelengths and 10 photo detectors on the flexible headband sensor. The configuration of light source and detectors yielded to a total of 16 active channels that composed the probe covering a space of 14.1 cm (width) by 3.5 cm (height). Such a system was designed and previously employed to monitor dorsal and inferior frontal cortical regions underlying the forehead (e.g., Izzetoglu et al., 2005; Bunce et al., 2006; Ayaz et al., 2010, 2012a,b,c) (see Figure 1A). Cognitive Optical Brain Imaging (COBI) Studio software (Drexel University) was used for data acquisition and visualization (Ayaz et al., 2011). During the task, a serial cable between the fNIR data acquisition computer and stimulus presentation computer was used to synchronize the fNIR and kinematic signals.

### Experimental procedure

To become familiarized with the experimental device, the participants performed 20 practice trials as an orientation/familiarization phase (these trials were not included in the subsequent analyses). Before starting the experiment, a 10 s baseline was recorded while the participants were inactive and relaxed. This baseline was then employed to compute the changes in concentration of fNIR markers (e.g., oxygenated hemoglobin) used in the subsequent standardization of the fNIR data. Then, participants performed 20 trials (i.e., 1 block) under normal (i.e., without visual distortion) visual feedback of cursor movement (i.e., pre-exposure). The 26 participants were randomly assigned to the learning and control groups with each group including 13 participants. The experimental (learning) group participants performed 180 (i.e., 9 blocks × 20 trials) drawing movements during which the screen cursor was suddenly rotated 60° counterclockwise (i.e., exposure) whereas no visual distortion was imposed on the control group. Lastly, all participants executed a block of 20 trials under normal visual feedback (i.e., no visual distortion) to assess for after-effects (i.e., post-exposure) and to determine if the internal model of the novel visuomotor map was effectively encoded by participants. Targets were self-selected and movements were self-initiated (i.e., no forced paced), and all targets were displayed throughout each trial. At the beginning of each trial, an 8 s rest period was considered during which the subject fixated on the home target (Sitaram et al., 2007). Then a beep sounded, indicating to the participants that they were allowed to enter the screen cursor inside the home target circle (without any time constraints). Next, participants had to select one of the four peripheral targets without moving the pen and prepare their movement. Once the peripheral target was selected, they had to draw a line as straight and as fast as possible linking the home and pointing target. Movements that started earlier than 2 s after target presentation were terminated, and the trial was restarted. Thus, participants had enough time to both select a target and plan their movement (at least 2 s) and could start whenever they felt ready after this 2 s period. Once a successful trial was performed, all visual stimuli were erased from the screen in preparation for the next trial. To minimize fatigue and maintain attention, brief relaxation periods were allowed as needed (Figure 1B).

### Data analysis

#### Movement kinematics

The kinematics of the hand were low-pass filtered using a 5-Hz, dual-pass eighth-order Butterworth filter. Then, to quantify the motor performance, three kinematic parameters were computed. Movement time (MT) defined as the time elapsed between leaving the home target and acquiring the reaching target and that reflected regulations during movement performance. Movement length (ML) represented the distance traveled in each trial between the home and the reaching target. Finally, root mean squared error (RMSE) was computed to quantify any discrepancy between the movement trajectory and the “ideal” straight line linking the home and the reaching target. After resampling of the trajectories to reach an equal amount of data points between the actual and “ideal” straight trajectory, the RMSE was computed according to the following formula:

(1) $$\text{RMSE}(\text{in cm})=\sqrt{\frac{\sum_{i=1}^{N}\left[{\left(x_{a}-x_{i}\right)}^{2}+{\left(y_{a}-y_{i}\right)}^{2}\right]}{N}}$$

where *x*_*a*_, *y*_*a*_, and *x*_*i*_, *y*_*i*_ are corresponding points of the actual (index *a*), resampled trajectory and the ideal (index *i*) trajectory, respectively. *N* is the number of points in the path.

In addition, a measure of directional error labeled initial directional error (IDE) was computed as the difference between the angle formed by the vector from the home position to the current hand position 80 ms after movement onset and the vector extending from the home position to the goal target (target to reach). Since this directional error is measured before visual feedback is available, measurement error information can inform about planning processes and the current state of the internal model of the perturbation. The kinematic parameters (MT, ML, and RMSE) and the directional error (IDE) were standardized with respect to the pre-exposure stage for each participant to account for any differences in a participants' performance during the pre-exposure stage (i.e., without perturbation) as well as to focus on changes that are solely due to adaptation. The values were standardized according to the following equation:

(2) $$SP_{i}(SD)=\frac{P_{i}-\bar{P_{\mathrm{Pr}\_Exp}}}{SDP_{\mathrm{Pr}\_Exp}}$$

where *P*_*i*_ (*P*: Parameter) is the value of a kinematic parameter computed for the *i*th single trial performed during exposure, and $\bar{P_{\mathrm{Pr}\_Exp}}$ and *SDP*_Pr_*Exp*_ represent the mean and standard deviation across trials of the same parameter computed during the pre-exposure block, respectively. The *SP*_*i*_ (*SP*: Standardized Parameter) values were then averaged within blocks and participants. As such, a standardization process was applied to the kinematic data, which were expressed in standard deviation units (i.e., *SD* units) relative to the pre-exposure stage for each participant.

#### fNIR signal processing

For each participant, raw fNIR data (16 optodes × 2 wavelengths) were low-pass filtered with a finite impulse response, linear phase filter with an order 20 and cut-off frequency of 0.1 Hz to attenuate the high frequency noise, respiration, and cardiac cycle effects (Izzetoglu et al., 2005; Ayaz et al., 2011). To check for any saturation, in which light intensity at the detector was higher than the analog-to-digital converter limit or motion artifact, both visual inspection and sliding window motion artifact rejection technique was used (Ayaz et al., 2010). fNIR data epochs for the baseline and task periods were extracted from the continuous data using time synchronization markers. Blood oxygenation and volume changes within each of the 16 optodes were calculated using the modified Beer-Lambert Law (Chance et al., 1993; Villringer and Chance, 1997) for task periods with respect to the baseline at beginning of the experiment with fnirSoft (Ayaz, 2010). For each task period, concentration changes of four parameters were calculated: oxygenated-hemoglobin (HBO2), deoxygenated hemoglobin (HB), total hemoglobin (HBT), and oxygenation (OXY—defined as the difference between HBO2 and HB). In order to ensure consistency in our data processing, the approach used for fNIR values was similar to that employed for kinematic parameters and error measurement. The fNIR values were also standardized by employing Equation 2. Then, the first eight optodes (1–8) were averaged to represent the left hemisphere while the last eight optodes (9–16) were average to represent the right hemisphere within the prefrontal cortex.

#### Statistical procedures and data fitting

Given that the purpose of this study was to investigate the relationship between cortical hemodynamics and performance by replicating a study using EEG power values and a visuomotor adaptation task (Gentili et al., 2011), the statistical plan was similar.

#### Statistical procedure for kinematic parameters

The average standardized values of the kinematic parameters (MT, ML, and RMSE) were assessed regarding meeting the parametric assumptions of normality using a *Kolmogorov-Smirnov test using a Lilliefors correction* as well as histograms. To assess the behavioral efficacy of the adaptation 2 × 2 Group (Learning and Control) by Period (Early and Late adaptation periods) mixed model ANOVAs with repeated measures on the last factor were computed separately for the kinematic parameters (MT, ML, RMSE) and the directional error (IDE). Adaptation periods were defined as early- (the two first blocks) and late- (the last two blocks) of task performance. The choice of the definition of the period for the early and late period was guided by previous studies that defined these periods in a similar way (e.g., Anguera et al., 2009; Gentili et al., 2011). Significance criterion for all tests was 0.05, percent change and 95% confidence intervals of the mean differences were calculated and presented in Table 1. For significant effects, partial omega-squared (ω^2^) is the effect size index presented for the data interpretation. Number Cruncher Statistical System (NCSS) 8 (www.ncss.com) software was used for the statistical analyses.

**Table 1:** **Descriptive statistics of the standardized kinematic parameters and the directional error across the groups including 95% confidence intervals**.

**Performance**	**Group**	**Mean ± SE (Early)**	**Mean ± SE (Late)**	**% Change**	**Mean difference value**	**Confidence interval (Lower limit)**	**Confidence interval (Upper limit)**
MT	EXP	5.58 ± 0.70	1.20 ± 0.33	78.49	4.56	3.11	6.01
MT	CON	−0.31 ± 0.08	−0.46 ± 0.16	48.39	0.15	−0.14	0.43
ML	EXP	7.85 ± 1.58	2.57 ± 0.74	67.26	5.28	2.91	7.66
ML	CON	0.16 ± 0.12	0.08 ± 0.14	50.00	0.07	0.12	−0.20
RMSE	EXP	8.16 ± 1.55	3.97 ± 1.11	51.35	4.19	2.40	5.97
RMSE	CON	0.30 ± 0.25	0.05 ± 0.10	83.33	0.25	−0.19	0.69
IDE	EXP	−2.35 ± 1.73	−0.80 ± 0.69	65.96	1.55	−2.62	−0.48
IDE	CON	−0.15 ± 0.24	−0.22 ± 0.31	51.94	−0.08	−0.15	0.30

MT, movement time; ML, movement length; RMSE, root mean squared error; IDE, initial directional error; EXP, experimental; CON, control; SE, standard error.

#### Statistical procedure for the fNIR parameters

A 2 × 2 × 2 [Group (learning; control) × Hemisphere (right; left) × Period (early; late)] mixed model ANOVA with repeated measures on the last two factors was applied separately to the four fNIR markers. The between subjects factor, Group, and the within subjects factors of Hemisphere and Period were fixed factors while the subject factor was a random effect. A Huynh–Feldt correction was applied (Huynh and Feldt, 1976) when the assumption of sphericity was violated. Any significant interaction effects were assessed by Tukey HSD tests for interactions. Cohen's d effect sizes were also computed and used to aid in data interpretation.

#### Data fitting of fNIR parameters

To characterize the cortical dynamics throughout the adaptation stage, the fNIR parameters were fitted throughout the nine practice blocks. A visual inspection of the fNIR data clearly suggested considering a linear fit. Thus, for the left and the right hemisphere and across the adaptation task, the changes in standardized fNIR parameters were fitted using a linear model for each participant. For each linear-fitted model, the coefficient of determination (*r*^2^) and its slope were obtained. Then, for each of the standardized fNIR parameters, the slopes of the linear models were statistically tested, as for the kinematic parameters, by employing a *Kolmogorov–Smirnov test using a Lilliefors correction* and histograms. A non-parametric *Wilcoxon tes*t was used to compare if the model values were statistically different (1) from 0 (i.e., if these models revealed a significant decrease or increase) and (2) between the participants of the learning and control group (i.e., if the dynamics of these model revealed a significant difference between the learning and the control group).

#### Relationship between cortical dynamics and kinematics parameters

Finally, to more directly examine the relationships between performance and the cortical hemodynamics, the values for the significant fNIR (HBO2, OXY) parameters were plotted as a function of the kinematics (MT, ML, and RMSE) for the left and the right hemispheres. A visual inspection of the data suggested four main possible fitting curves: a linear [*f*(*x*) = *ax* + *b* {*a*, *b*} ∈ *R*], a logarithmic [*f*(*x*) = *a* log (*x*){*a*} ∈ *R*], a rational $\left[f(x)=\frac{a}{x}\{a\}\in R\right]$, and a composite function that combined a rational function and a linear component $\left[f(x)=\frac{a}{x}+bx+c\{a,b,c\}\in R\right]$. The best fit was selected by considering *r*^2^ of the fit.

## Results

### Movement kinematics

**Figure 2:**
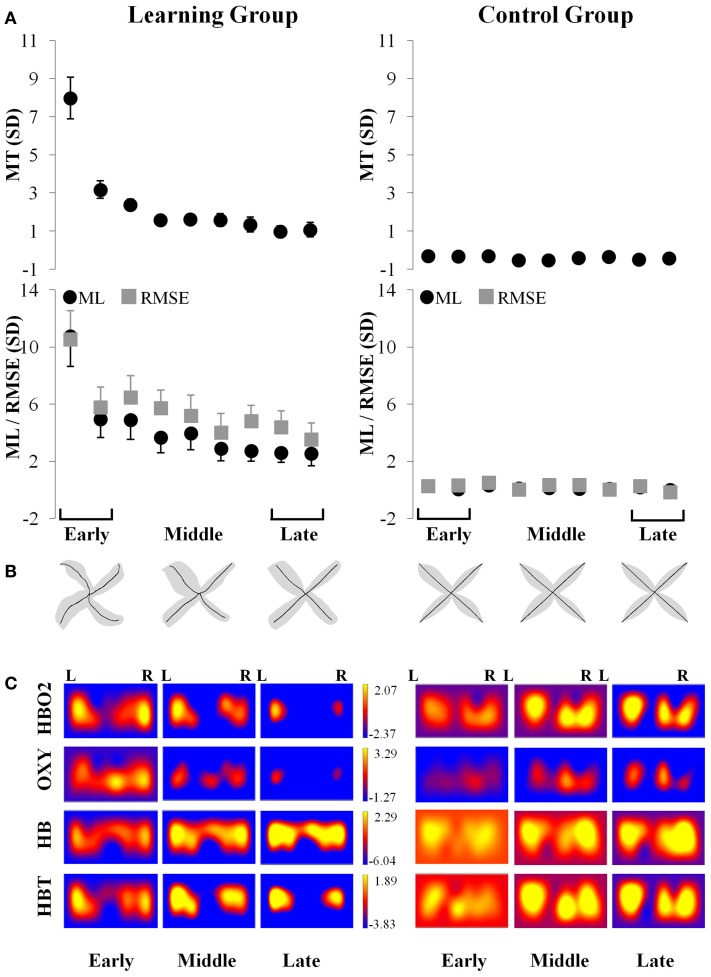
**Simultaneous kinematic and fNIR changes during adaptation for the learning (left column) and control (right column) groups. (A)** Changes in movement time (±SE), movement length (±SE; filled black circle) and root mean squared error (±SE; filled gray square) throughout the practice blocks. **(B)** Changes in average trajectory (thick black lines) throughout adaptation for early, middle, and late exposure (the gray area illustrates the standard error across subjects). **(C)** Changes in the magnitude throughout adaptation for early, middle, and late exposure of the standardized fNIR hemodynamics including HBO2 (first row); OXY (second row); HB (third row); and HBT (fourth row) in sd units for the left and right prefrontal regions. MT, movement time; ML, movement length; RMSE, root mean squared error; R, Right; L, Left.

During early exposure to the visuomotor perturbation the participants of the learning group revealed movement similar to counter-clockwise spirals trajectories that included sudden reversals and slow progression toward the targets whereas during the late exposure stage movement trajectories were faster, straighter, and with a reduction of the RMSE as noted in Table 1. Movement kinematics resulted in significant differences for the Group × Period interaction, Period main effect and Group main effects which are reported in Table 2. The significant Group × Period interactions and Period main effects revealed that, compared to early adaptation, MT, ML, RMSE, and initial directional error were reduced during the late-exposure stage (see Figures 2A,B; left column). In addition, a very large effect ω^2^_partial_ = 0.37−0.64 was detected for the movement kinematics as a function of the interactions or Period main effects. During the post-exposure stage, (i.e., once the distortion was removed), movement trajectories showed distortions (after-effects) with movements in the opposite direction compared to the early stages of adaptation revealing the participants had encoded the internal model of the new visuomotor transformation. Conversely, participants of the control group did not reveal any changes in performance throughout the entire task as suggested by the absence of changes in the hand paths (see Figures 2A,B; right column).

**Table 2:** **Results of the mixed model ANOVAs (2 Group × 2 Period) for the kinematic parameters and the directional error obtained for the learning and control groups during the early and late adaptation periods**.

**Performance**	**Effect**	***F*_(1, 24)_ =**	***p*-value**	**Effect size index ωpartial^2^/ω^2^**
MT	Group × Period	42.26	<0.001	0.61
	Period	48.11	<0.001	0.64
	Group	68.81	<0.001	0.72
ML	Group × Period	22.58	<0.001	0.49
	Period	23.89	<0.001	0.50
	Group	20.91	<0.001	0.43
RMSE	Group × Period	21.83	<0.001	0.48
	Period	27.70	<0.001	0.54
	Group	20.60	<0.001	0.43
IDE	Group × Period	19.50	<0.001	0.42
	Period	15.98	<0.001	0.37
	Group	18.14	<0.001	0.40

MT, movement Time; ML, movement length; RMSE, root mean squared error; IDE, initial directional error. ω^2^, effect size index (proportion of variance explained) for between subjects main effect; ω partial^2^, effect size index for interactions and within subjects main effect.

### fNIR value: early (first two) vs. late (last two) blocks of trials

A 2 × 2 × 2 ANOVA (Group × Hemisphere × Period) was applied separately to the four fNIR markers (i.e., HBO2; OXY; HBT; and HB). Significance criterion for all tests was 0.05 and 95% confidence intervals of the mean differences and percent change were calculated and presented in Table 3.

**Table 3:** **Descriptive statistics of the standardized hemodynamic parameters across the learning and control groups and prefrontal sides including 95% confidence intervals**.

**fNIR Parameter**	**Prefrontal side**	**Group**	**Mean ± SE (Early)**	**Mean ± SE (Late)**	**% Change**	**Mean difference value**	**Confidence interval (Lower limit)**	**Confidence interval (Upper limit)**
HBO2	Left	EXP	−0.28 ± 0.52	−4.20 ± 1.28	93.33	3.92	1.87	5.98
HBO2	Right	EXP	−0.02 ± 0.75	−4.41 ± 1.16	99.55	4.39	2.48	6.30
HBO2	Left	CON	−0.09 ± 0.47	−0.80 ± 1.77	88.75	0.71	−2.51	3.94
HBO2	Right	CON	0.06 ± 0.38	−0.11 ± 1.80	45.46	0.18	−3.15	3.52
OXY	Left	EXP	0.76 ± 0.67	−4.01 ± 1.17	81.05	4.77	1.54	8.00
OXY	Right	EXP	1.29 ± 0.86	−3.90 ± 1.36	66.92	5.19	2.06	8.33
OXY	Left	CON	−0.47 ± 1.52	−1.10 ± -5.38	57.27	0.63	−3.02	4.27
OXY	Right	CON	−0.19 ± 0.41	−1.05 ± 1.94	81.90	0.85	−2.88	4.59
HB	Left	EXP	−1.84 ± 0.85	−1.29 ± 1.89	42.64	−0.55	−3.88	2.78
HB	Right	EXP	−1.82 ± 0.84	−0.51 ± 0.93	256.86	−1.31	−2.87	0.26
HB	Left	CON	0.51 ± 0.49	−0.67 ± 1.26	23.88	1.18	−0.94	3.30
HB	Right	CON	0.92 ± 0.50	2.05 ± 1.12	55.12	−1.12	−3.03	0.78
HBT	Left	EXP	−0.98 ± 0.55	−3.77 ± 1.35	74.01	2.80	0.15	5.44
HBT	Right	EXP	−1.22 ± 0.74	−3.94 ± 0.90	69.04	2.72	0.51	4.93
HBT	Left	CON	0.23 ± 0.52	−0.55 ± 1.57	58.49	0.78	−1.93	3.48
HBT	Right	CON	0.20 ± 0.32	0.60 ± 1.70	66.67	−0.41	−3.56	2.74

HBO2, oxygenated hemoglobin; OXY, oxygenation; HB, deoxygenated hemoglobin; HBT, total hemoglobin; EXP, experimental; CON, control; SE, standard error.

### HBO2

**Figure 3:**
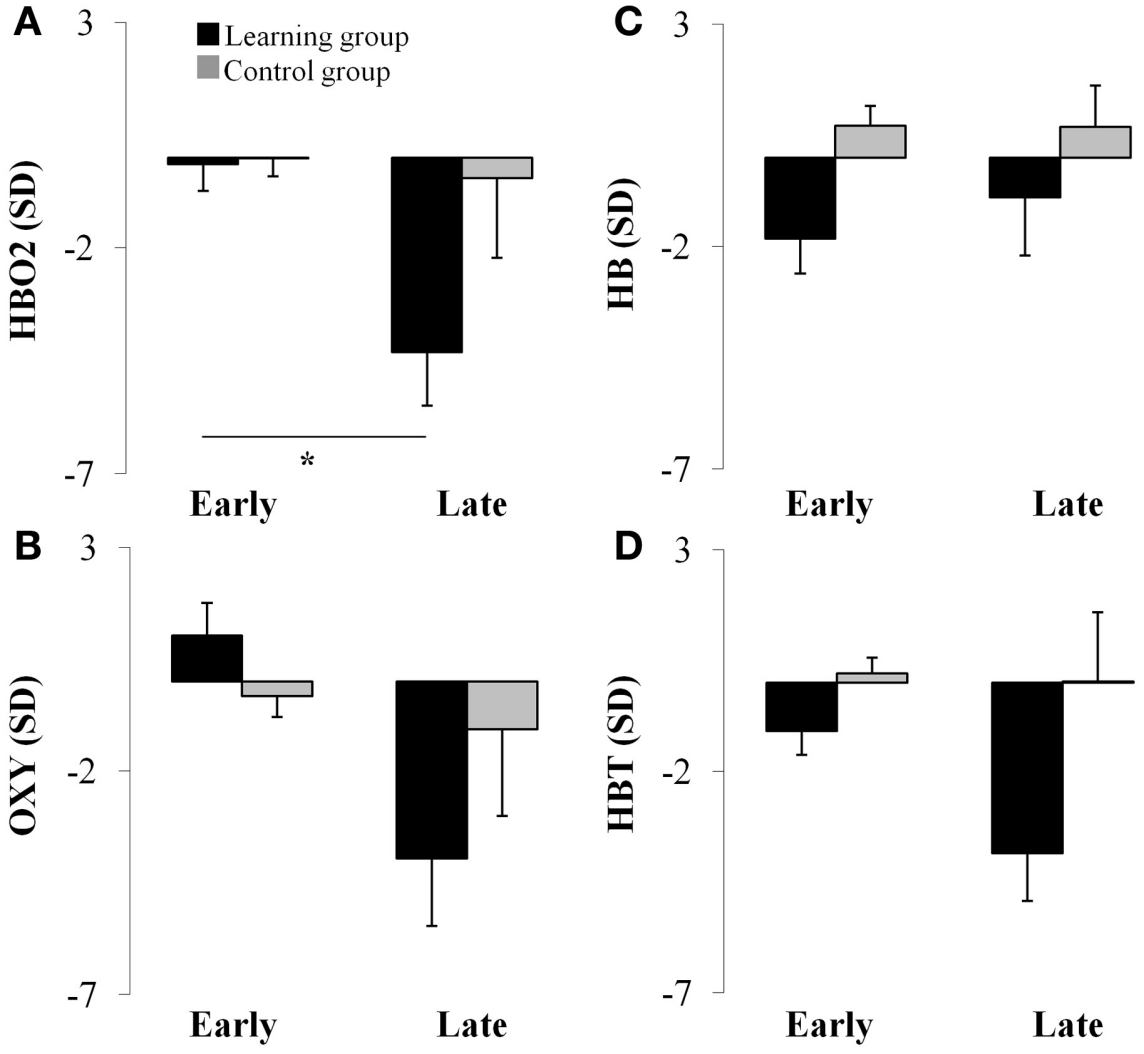
**Average hemodynamics (collapsed across hemisphere) for the early and late adaptation period for HBO2 (panel A), OXY (panel B), HB (panel C), and HBT (panel D) for the learning (black color) and control (gray color) groups.** ^*^p < 0.05.

The results of the ANOVA revealed a two-way interaction between Group and Period [*F*_(1, 24)_ = 4.67, *p* < 0.05] for the HBO2 marker. This analysis showed that HBO2 in the prefrontal region was significantly lower for the late (*M* = −4.31 sd units, *SE* = 1.19; *d* = 1.26) compared to the early (*M* = −0.15 sd units, *SE* = 0.59) adaptation phase in the learning group (*p* < 0.012) whereas no change was detected (*p* > 0.98) in the control group (Figures 2C, 3A).

### OXY

ANOVA revealed a Period main effect [*F*_(1, 24)_ = 6.66, *p* < 0.05] for the OXY marker suggesting that, compared to the late adaptation (*M* = −2.51 sd units, *SE* = 1.76, *d* = 0.66), the OXY was significantly higher during early adaptation stage (*M* = 0.35 sd units, *SE* = 0.65) for both groups. Interestingly, ANOVA revealed a tendency regarding a two-way interaction between Group and Period (*p* = 0.06) which suggest that the Period main effect was likely mainly driven by changes in the learning group. Although not significant, the OXY in the prefrontal region tended to be lower for the late (*M* = −3.95 sd units, *SE* = 1.52, *d* = 1.21) compared to the early (*M* = 1.03 sd units, *SE* = 0.76) adaptation phase in the learning group whereas the same comparison for the control group revealed a smaller difference (early: *M* = −0.33 sd units, *SE* = 0.46 vs. late: *M* = −1.03 sd units, *SE* = 1.91, *d* = 0.17; see Figures 2C, 3B). The high standard errors may have contributed to reducing the power of this test.

### HB

ANOVA did not reveal any effect (*p* > 0.05) for the HB marker suggesting that, both the learning and control group had a comparable level of HB throughout practice (see Figures 2C, 3C).

### HBT

ANOVA revealed a Group main effect [*F*_(1, 24)_ = 5.07, *p* < 0.05] for the HBT marker suggesting that during practice, in comparison to the control group (*M* = 0.12 sd units, *SE* = 4.21, *d* = 0.61), the HBT was lower in the learning (*M* = −2.48 sd units, *SE* = 0.98) group. In addition, the Period main effect exhibited a tendency in the same direction for the early and late phases which was comparable to the OXY and HBO2 biomarkers (*p* = 0.08). The high variability during the late phase contributed to the reduced statistical power and higher Type II error for this effect (see Figures 2C, 3D).

### Linear model of fNIR markers across all blocks of trials

**Figure 4:**
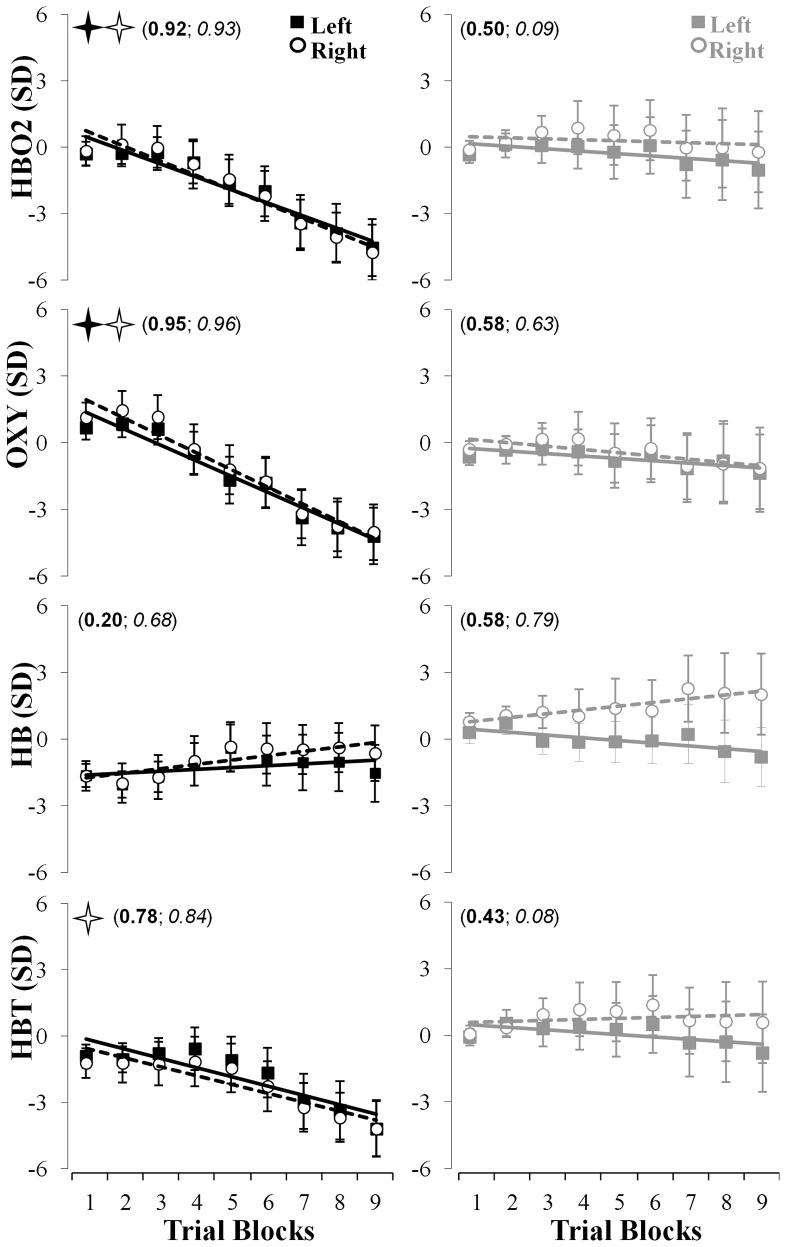
**Linear fits of the fNIR hemodynamics changes in the left (full squares, solid lines) and right (empty circles, dashed lines) prefrontal regions for HBO2 (first row), OXY (second row), HB (third row), and HBT (fourth row) throughout the nine blocks of adaptation.** The left (in bold) and right (in italic) numbers in parentheses represent the coefficients of determination for the left and right prefrontal regions, respectively. The full and empty black stars indicate that the slopes were significantly different from 0 for the left and right prefrontal regions, respectively. The left and right columns represent changes in the fNIR hemodynamics for the learning and control groups, respectively.

The data fitting approach (see Figure 4, top row) revealed that the linear fitting [*f*(*x*) = *ax* + *b* {*a*, *b*} ∈ *R*] model captured accurately the changes in hemodynamics indicated by the HBO2 marker and revealed a significant linear decrease in both the left (*r*^2^ = 0.92; *p*_*slope*_ < 0.002; *a* = −0.59; *b* = 1.07) and right (*r*^2^ = 0.93; *p*_*slope*_ < 0.001; *a* = −0.65; *b* = 1.41) hemisphere throughout the nine blocks of trials in the learning group whereas such change was not significant for the control group (*r*^2^ < 0.51; *p*_*slope*_ > 0.50; *a* > −0.11; *b* > 0.26). Also, compared to the control group, the linear decrease for the right hemisphere was significantly higher (*p* < 0.013) for the learning group whereas a tendency in a similar direction was observed for the left hemisphere (*p* = 0.06). The oxygenation level indicated by the OXY marker revealed a very large effects and significant linear decrease in both the left (*r*^2^ = 0.95; *p*_*slope*_ < 0.007; *a* = −0.71; *b* = 2.10) and right (*r*^2^ = 0.96; *p*_*slope*_ < 0.004; *a* = −0.78; *b* = 2.71) hemisphere throughout the nine blocks of trials in the learning group whereas the control group did not exhibit these changes (*r*^2^ < 0.63; *p*_*slope*_ > 0.57; *a* > −0.14; *b* > −0.14). Also, compared to the control group, the linear decrease for the right hemisphere revealed a tendency to be higher (*p* = 0.07) for the learning group (see Figure 4, second rows). The same linear modeling applied to the HB did not reveal any significant decrease or increase for both the learning and the control groups (0.20 < *r*^2^ < 0.79; *p*_*slope*_ > 0.20; *a* > −0.12; *b* > −1.67). This linear fit revealed a significant decrease of HBT in the right hemisphere for the learning group (*r*^2^ > 0.78; *p*_*slope*_ < 0.013; *a* = −0.41; *b* = −0.16) whereas no significant change was observed in the control groups (*r*^2^ < 0.43; *p*_*slope*_ > 0.54; *a* > −0.11; *b* > 0.60; see Figure 4, third and fourth rows).

### Relationship between fNIR parameters level and task performance

**Figure 5:**
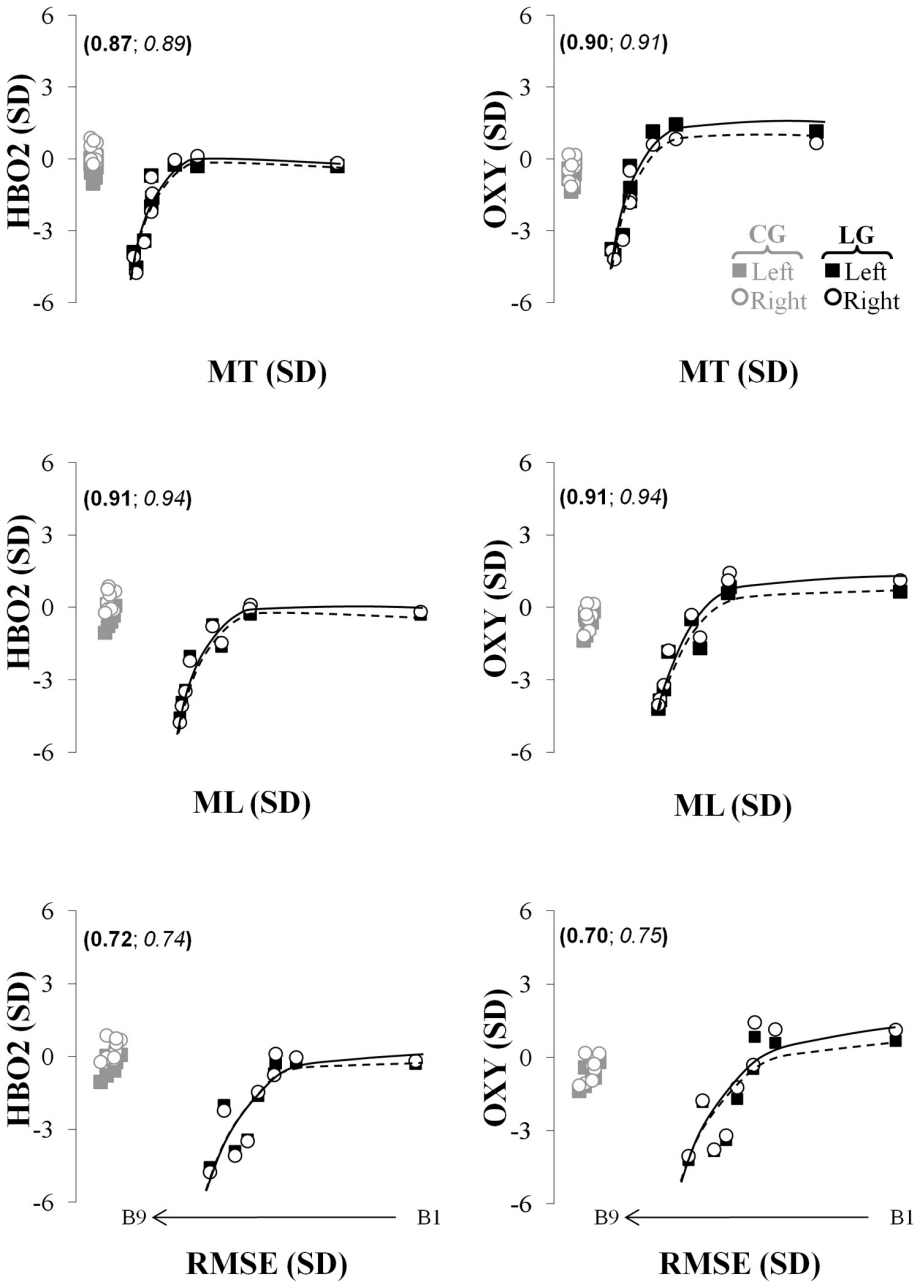
**Relationship capturing the concurrent changes in kinematics and fNIR hemodynamic in the left (full squares, solid lines) and right (empty circles and dashed lines) prefrontal regions for the HBO2 (first row), the OXY (second row).** The left (in bold) and right (in italic) numbers in parentheses represent the coefficients of determination of these relationships for the left and right prefrontal regions, respectively. The black and gray colors represent changes in the fNIR hemodynamics for the learning and control groups, respectively. The first, second, and third rows represent the relationship between the changes in the fNIR hemodynamics and the movement time, the movement length, and the root mean squared error, respectively. MT, movement time; ML, movement length; RMSE, root mean squared error; B, trial block.

To establish a more direct relationship between the observed changes in fNIR markers and kinematic performance throughout the visuomotor adaptation performance, correlational, and data fitting analyses were conducted. Generally, the relationship between the changes in oxygenation levels (i.e., HBO2, OXY) in both hemispheres and the kinematic parameters (MT, ML, RMSE) observed in the learning group was best modeled by means of the composite function (Figure 5). Specifically, this fitting model accurately captured the relationship between the HBO2 and the three kinematic parameters (*r*^2^ > 0.72; see Figure 5, left column) as well as between the OXY measurement and the three same kinematics parameters (*r*^2^ > 0.70; see Figure 5, right column) for the participants of the learning group. The coefficient of determination was very large and accounted for a substantial amount of the explained variance in the hemodynamic variables (HBO2 and OXY) as a function of the kinematic measures.

The model could not capture the same relationship considering HB (which actually presented an opposite directionality to the logarithmic function) whereas it could moderately capture the relationship between the changes in HBT and the three kinematics parameters for the learning group (0.38 ≤ *r*^2^ ≤ 0.48; not shown in Figure 5). Finally, the same analyses could not fit the relationship between the hemodynamic markers and the kinematics performance for the control group since no particular patterns was observed in the data. Rather the data represented a simple clustering effect [see Figure 5; the gray empty circles and filled squares for the HBO2 (right column) and OXY (left column)].

## Discussion

The main results of this investigation were the reduction of the hemodynamics and oxygenation level as indicated by the changes in HBO2 and to a lesser extent in OXY in the prefrontal regions as participants of the learning group progressively encoded the internal model of the new visuomotor transformation and thus improved their performance during the cognitive-motor adaptation task. Importantly, these reductions of oxygenation in the prefrontal regions were accompanied by a simultaneous decrease in variability and by a reduction in MT, ML, RMSE and directional error that resulted in performance enhancement. No changes either in brain hemodynamics or behavioral performance were observed in the participants of the control group who performed the same task as the learning group without any visuomotor distortion. Therefore, it appears reasonable to consider that the changes in oxygenation (HBO2 and OXY) observed in the prefrontal regions of the participants of the learning group were associated with adaptation of the prefrontal cortical dynamics that translated into enhanced quality of motor performance. Such changes in the learning group provide hemodynamic-based brain biomarkers (Georgopoulos et al., 2007; Gentili et al., 2009, 2010a,b, 2011) that can be employed to track the state of motor adaptation and more generally the changes in quality of performance.

### Frontal executive functioning for adaptive cognitive-motor challenge

As expected, a reduction of the oxygenation in the prefrontal regions was observed throughout adaptation. A decrease in oxygenation reflects a progressive reduction of activation of these prefrontal regions suggesting that there is a reduction of the role of the prefrontal cortex as adaptation progresses. Specifically, the highest magnitude of oxygenation was observed during early adaptation suggesting an initial pronounced engagement of these prefrontal regions that was progressively reduced as participants adapted to the task demands. This evidence was also supported by the non-standardized values of HBO2 and OXY (not reported here) which, although similar during the pre-exposure session, suddenly increased when the perturbation was introduced and then decreased as adaptation progressed. Specifically, compared to the control group a 65% and a 82% larger increases for the HBO2 and OXY markers were observed for the learning group, respectively. Furthermore, the effect size for the early to late oxygenation transition for the learning group was large (*d* = 1.21) while the control group had a small to negligible effect (*d* = 0.17). These findings support our hypothesis about the differential involvement of the frontal executive functioning during early and late adaptation to a new visuomotor transformation. More precisely, while the prefrontal regions play a critical role in multiple neural processes, a possible explanation for this gradual prefrontal derecruitment would be related to the executive functioning processes and, specifically, to the inhibitory and the updating functions. Thus, during early exposure the introduction of the sudden kinematic perturbation challenged the individuals of the learning group since they are now facing a new visuomotor map: (1) for which their prepotent visuomotor transformation (i.e., their usual motor response in absence of perturbation) becomes suddenly irrelevant and (2) that creates a mismatch between the visual feedback related to the cursor trajectory and the kinesthetic signals from the arm/hand movement, resulting in sensorimotor conflicts and poor performance. Thus, during early exposure, the inhibitory function would play a critical role by inhibiting the unsuitable prepotent visuomotor transformation. This inhibition would allow for an efficient adaptation (e.g., Miller and Cohen, 2001; Basso et al., 2006) by facilitating the selection process of a new and well-suited visuomotor plan to solve sensorimotor conflicts and meet the task requirements (i.e., move the cursor as straight and fast as possible). Concurrently, the frontal updating function would constantly update working memory by evaluating newer incoming external (visual input) and internal (kinesthetic) information (Miyake et al., 2000; Shimamura, 2000). It was previously suggested that both functions could be implemented in the prefrontal regions (Collette and Van der Linden, 2002), and specifically, in the dorsolateral prefrontal and frontopolar cortices which are used to evaluate externally (visual, kineasthetics) and internally (motor command) generated information during movement preparation (Christoff and Gabrieli, 2000; Braver and Bongiolatti, 2002), respectively. Over time, as the participant adapts, the role of these inhibitory and updating functions progressively decreases, resulting in a gradual deactivation (reflected by a reduction of the oxygenation level) of the prefrontal regions by late exposure.

Importantly, by employing an alternative neuroimaging methodology, the present findings confirm and extend those from previous EEG and fMRI studies that revealed an increased role of frontal and prefrontal (dorsolateral, ventral) regions during early compared to late visuomotor adaptation and particularly underscore the role of the frontal executive (inhibitory, updating functions) when a new visuomotor transformation is being encoded (e.g., Shadmehr and Holcomb, 1997, 1999; Ghilardi et al., 2000; Graydon et al., 2005; Lacourse et al., 2005; Anguera et al., 2007; Gentili et al., 2010a, 2011). In a previous study, Gentili et al. (2011) analyzed EEG and kinematics using exactly the same visuomotor task including a learning group where participants had to adapt to a new visuomotor transformation and a control group who performed the same visuomotor task without any perturbation. The results of the Gentili et al. (2011) EEG study reported a bilateral increase in alpha power in the prefrontal regions that translated into the improvement of the quality of performance as individuals of the learning group adapted to the task demands. By considering that the alpha power is inversely related to brain activation, thus, an increase in EEG alpha power reflects a progressive cortical idling or in other words a progressive refinement of the cortical activity (Pfurtscheller et al., 1996; Hatfield et al., 2004). Therefore, in the present investigation, the reduction of oxygenation level observed bilaterally in the prefrontal regions is consistent with the reduction of the cortical activity previously reported by an increase in EEG alpha power. In addition, the findings of the Gentili et al. (2011) EEG study also revealed that no change either in performance or alpha power (i.e., no change in cortical activation) was observed in participants of the control group. This finding is also consistent with the present results for the control group where no substantial change either in performance or in the oxygenation level (and thus in cortical activation) was observed. Although the limited spatial resolution of EEG and fNIR do not allow to accurately identify which brain regions would implement such inhibitory function, our results are in accordance with a previous fMRI study that employed a similar adaptation task and suggested the involvement of the ventral prefrontal cortex during inhibition of competing internal models of visuomotor transformations (Shadmehr and Holcomb, 1999).

In addition to these EEG and fMRI studies, and although still relatively rare, the few fNIR learning studies provide a developing body of evidence that cortical hemodynamics change as a function of learning new motor skills and practice (Hatakenaka et al., 2007; Ikegami and Taga, 2008; Leff et al., 2008a,b; Morihiro et al., 2009; Gentili et al., 2010a,b). Particularly, in agreement with our results, a reduction of cortical hemodynamics was revealed in the prefrontal cortex throughout practice while performance was enhanced (Leff et al., 2008a,b; Ohuchida et al., 2009; Ayaz et al., 2012a). The authors suggested that such attenuation would reflect a refinement of the prefrontal regions activity involved in executive functioning and particularly those related to attentional processes as well as to working memory supervised by the updating function in order to encode new spatiotemporal arrangements. The observed changes in cortical hemodynamics during this adaptation task could also reflect changes in attentional processes that were previously identified as common practice effects in various studies examining procedural skills learning (e.g., Leff et al., 2008a,b, 2011; Ohuchida et al., 2009). Importantly, this change in cortical hemodynamics could provide a complementary explanation to the procedural skills attention processes that are related to inhibitory control. During early adaptation, the attentional resources are largely depleted by the task. This depletion of resources occurs in conjunction with the need to inhibit familiar responses, however when performance becomes more automatic during late adaptation the attentional resources are regained.

Also, it must be noted that the refinement of the cortical hemodynamics (HBO2, OXY) and kinematics have different time scales since they follow linear and non-linear (exponential) dynamics, respectively. Such time-scale discrepancies between cortical hemodynamic and kinematics were also observed in previous EEG studies that used the same task (Gentili et al., 2009, 2011; Rietschel, 2011). A possible explanation for the time-scale discrepancies would be that, although performance strongly improves over a certain number of trials, the brain is still engaged in a substantial effort to perform the task successfully and thus a high degree of cortical activity is observed. Although, at some point, additional practice does not necessarily result in a substantial improvement of the behavioral performance, the additional practice definitely contributes to enhancing the automaticity of performance that translates into continuous refinement of the prefrontal activity and thus into a continuous reduction of the oxygenation level (Rietschel, 2011). With additional practice, we contend that the prefrontal hemodynamics would also reach an asymptotic level.

Nevertheless, it must be noted that a prefrontal asymptotic leveling response was not systematically observed in experiments that involved motor practice. For instance, during a rotor pursuit practice task, while a reduction in the activation of the pre-supplementary motor area was observed, no particular hemodynamic change was revealed in the prefrontal cortex (Hatakenaka et al., 2007). Such discrepancy may be due to differences in experimental paradigms and to the nature and/or the demands of the task. Contrary to our task, practice of the pursuit rotor task (Hatakenaka et al., 2007) required mainly refining existing motor commands without the need to inhibit prepotent motor plans that could interfere with task performance.

Therefore, the high magnitude of activation in the prefrontal regions during early learning would be primarily related to executive functioning and particularly to the updating function to appraise working memory and inform changes in attentional resources along with the inhibitory function to suppress prepotent motor responses of inappropriate actions. The role of such frontally mediated functions is reduced during late learning and thus leads to a smaller activation of these prefrontal regions (Ghahremani et al., 2010). Overall, our results confirm and extend those from previous studies employing various neuroimaging techniques (e.g., fMRI, PET, EEG, fNIR) as well as reinforces that idea that the frontal executive has not only a critical role for cognitive control involved in purely cognitive tasks (e.g., Stroop task, Collette et al., 2006), but also for cognitive-motor/sensorimotor learning challenges, contributing to bridging the gap between the cognitive and cognitive-motor/sensorimotor control fields. It must be noted that the current study employed a fNIR probe that only covered the forehead. Thus, although the use of this technique was guided by our hypothesis, additional research is needed by employing a whole head fNIR system in order to further examine the hemodynamic responses of other cortical regions during adaptation.

### Hemodynamics-based brain biomarkers for ecologically valid cognitive-motor performance

To our knowledge, the present study is the first fNIR investigation of a visuomotor adaptation task where the participants need to inhibit prepotent motor plans while performing multi-joint arm reaching movements from a seated position allowing certain latitude in term of mobility. The present study extends and confirms the notion that fNIR technology allows for recording and analyzing the neural activity during cognitive-motor performance and learning task in ecologically valid situations where individuals can be seated rather than constrained to a supine position as is the case when employing fMRI (Hatakenaka et al., 2007; Ikegami and Taga, 2008; Ayaz et al., 2009, 2011, 2012a,b,c; Morihiro et al., 2009; Gentili et al., 2010a,b). In addition, this type of signal may be more resilient to noise compared to EEG such as less susceptibility for artifacts from eye-movements, muscular activity, and surrounding electrical interferences (Coyle et al., 2004; Sweeney et al., 2012) and therefore also contribute to reinforce its suitability for applications in the field. From an applied perspective, cortical hemodynamics may play a significant role in a broad range of applications in the field of operational neurosciences. For instance, these hemodynamic-based biomarkers may be useful for monitoring brain activity during ecologically valid adaptive movements where upper limbs are involved in learning/re-learning a motor task or adapting to a new tool/environment in rehabilitation and/or a human factors context. Besides the critical advantage of fNIR to perform a task in an ecologically valid environment, another important advantage of fNIR over fMRI is that additional biomarkers can be derived from the information related to hemodynamic responses which include HBO2, OXY, and HBT and not just HB (Leff et al., 2011; Ayaz et al., 2012a). Although multiple hemodynamic markers can be derived from fNIR, Leff et al. (2011) noted that many fNIR studies only considered one hemodynamic marker to examine brain activation (Miyai et al., 2003; Suzuki et al., 2004; Takeda et al., 2007; Ohuchida et al., 2009). We are in agreement with reporting multiple hemodynamic markers in fNIR studies and thus, the current investigation assessed HBO2, HB, and HBT, as well as the OXY marker which reflected the level of total oxygenation.

However, it is also important to note that despite its numerous advantages, fNIR also has important limitations such as a limited temporal resolution compared to other techniques (e.g., EEG). This is an important limitation since such reduced temporal resolution does not allow investigators to examine separately the planning and execution phase as previously done with studies using EEG (Gentili et al., 2009, 2011). Another limitation of fNIR is its sensitivity to head orientation since this can change the blood flow and thus impact the fNIR signals irrespective of the task effects and of the multiple existing approaches developed to eliminate such artifacts (Boas et al., 2001; Zhang et al., 2009; Ayaz et al., 2010; Izzetoglu et al., 2010; Sweeney et al., 2012; Umeyama and Yamada, 2013). Also, the spatial resolution of spectroscopy-based systems is limited in the optode screening depth to half of the distance between the light source and detector (Strangman et al., 2002). Therefore, both fNIR and EEG techniques appear to be complementary techniques that could be combined in order to provide multi-modal hemodynamic and electrical-based brain markers.

Interestingly, as described earlier, the results obtained in this fNIR study are consistent with those previously obtained employing EEG with exactly the same task (Gentili et al., 2011). Although no co-registration of fNIR and EEG was performed, the strict parallel of this experimental protocol and that employed by Gentili et al. (2011) contributes to reinforce the idea to combine these two neuroimaging approaches. One important advantage of combining these two neuroimaging technologies would be to have access to multiple markers derived from both electrical activity and hemodynamic responses that act at different temporal and spatial scales. This would be particularly helpful for investigating the brain dynamics and more generally for brain monitoring applications to accurately assess the level of cognitive-motor performance (Gentili et al., 2010a,b). Because the underlying physics principles of these multimodal technologies, which may be attributed to the fact that the light signal does not interfere with electrical or magnetic fields (Coyle et al., 2007), we posit that a combination of fNIR and EEG or fNIR and fMRI seem plausible. Thus, a system combining both EEG and fNIR technologies could be deployed in the field with the possible capabilities to provide different and complementary brain biomarkers that can be used to robustly investigate brain functioning in ecologically valid, naturalistic situations (Coyle et al., 2007; Roche-Labarbe et al., 2007; Gentili et al., 2010b).

## Conclusion

This was the first study to investigate adaptive arm reaching movement employing fNIR technology. Specifically, the findings revealed that throughout adaptation to a new visuomotor transformation, it was possible to derive fNIR-based hemodynamic markers in terms of oxygenation levels (HBO2 and OXY) in the prefrontal regions to assess the ongoing progression of the adaptation processes. Our findings are supported by previous EEG results obtained by employing exactly the same reaching task adaptation paradigm. The study confirms the previously proposed principle that the refinement of the cortical dynamics during adaptation translate into the quality of the motor performance. More precisely, the gradual reduction of oxygenation in the prefrontal regions observed throughout adaptation suggest a pronounced initial involvement of frontal executive processes such as inhibitory and updating functions that is progressively derecruited as the internal model of the new visuomotor transformation is gradually encoded and the task is mastered. The present findings contribute to expand our understanding of the role of frontal executive functioning beyond the purely cognitive field to the cognitive-motor/sensorimotor control field. Overall the observed changes in the cortical hemodynamics represent potential brain biomarkers (Georgopoulos et al., 2007; Gentili et al., 2010a,b, 2011) that could be combined with different and complementary EEG markers (Gentili et al., 2011) to assess the level of adaptive cognitive-motor performance.

### Conflict of interest statement

The authors declare that the research was conducted in the absence of any commercial or financial relationships that could be construed as a potential conflict of interest.

## References

[B1] Abdelnour A. F. Huppert T. (2009). Real-time imaging of human brain function by near-infrared spectroscopy using an adaptive general linear model. Neuroimage 46, 133–143 10.1016/j.neuroimage.2009.01.033 19457389 PMC2758631

[B2] Anguera J. A. Russell C. A. Noll D. C. Seidler R. D. (2007). Neural correlates associated with intermanual transfer of sensorimotor adaptation. Brain Res. 14, 136–151 10.1016/j.brainres.2007.09.088 17996854

[B3] Anguera J. A. Seidler R. D. Gehring W. J. (2009). Changes in performance monitoring during sensorimotor adaptation. J. Neurophysiol. 102, 1868–1879 10.1152/jn.00063.2009 19605614 PMC2746769

[B4] Ayaz H. (2010). Functional Near Infrared Spectroscopy based Brain Computer Interface. Ph.D. thesis, Drexel University, Philadelphia, PA.

[B5] Ayaz H. Shewokis P. A. Bunce S. Izzetoglu K. Willems B. Onaral B. (2012a). Optical brain monitoring for operator training and mental workload assessment. Neuroimage 59, 36–47 10.1016/j.neuroimage.2011.06.023 21722738

[B6] Ayaz H. Bunce S. Shewokis P. A. Izzetoglu K. Willems B. Onaral B. (2012b). Using brain activity to predict task performance and operator efficiency, in Advances in Brain Inspired Cognitive Systems, eds Zhang H. Hussain A. Liu D. Wang Z. (Heidelberg: Springer), 147–155

[B7] Ayaz H. Shewokis P. A. Ýzzetoğlu M. Çakır M. P. Onaral B. (2012c). Tangram solved? Prefrontal cortex activation analysis during geometric problem solving, in Proceedings of the 34th Annual International Conference of the IEEE Engineering in Medicine and Biology Society (EMBS'12), (San Diego, CA), 4724–4727 10.1109/EMBC.2012.6347022 23366983

[B8] Ayaz H. Izzetoglu M. Shewokis P. A. Onaral B. (2010). Sliding-window Motion Artifact Rejection for Functional Near-Infrared Spectroscopy. Conf. Proc. IEEE Eng. Med. Biol. Soc. 6567–6570 10.1109/IEMBS.2010.5627113 21096508

[B9] Ayaz H. Shewokis P. A. Curtin A. Izzetoglu M. Izzetoglu K. Onaral B. (2011). Using MazeSuite and functional near infrared spectroscopy to study learning in spatial navigation. J. Vis. Exp. 56:e3443 10.3791/3443 22005455 PMC3227178

[B10] Ayaz H. Shewokis P. Bunce S. Schultheis M. Onaral B. (2009). Assessment of cognitive neural correlates for a functional near infrared-based brain computer interface system. Found. Augment. Cogn. Neuroergonom. Oper. Neurosci. 5638, 699–708 10.1007/978-3-642-02812-0_79

[B11] Basso D. Lotze M. Vitale L. Ferreri F. Bisiacchi P. Olivetti Belardinelli M. (2006). The role of prefrontal cortex in visuo-spatial planning: a repetitive TMS study. Exp. Brain Res. 171, 411–415 10.1007/s00221-006-0457-z 16710684

[B12] Boas D. A. Gaudette T. Strangman G. Cheng X. Marota J. J. Mandeville J. B. (2001). The accuracy of near infrared spectroscopy and imaging during focal changes in cerebral hemodynamics. Neuroimage 13, 76–90 10.1006/nimg.2000.0674 11133311

[B13] Bradberry T. J. Rong F. Contreras-Vidal J. L. (2009). Decoding center-out hand velocity from MEG signals during visuomotor adaptation. Neuroimage 47, 1691–1700 10.1016/j.neuroimage.2009.06.023 19539036

[B14] Braver T. S. Bongiolatti S. R. (2002). The role of frontopolar cortex in subgoal processing during working memory. Neuroimage 15, 523–536 10.1006/nimg.2001.1019 11848695

[B15a] Bunce S. C. Izzetoglu M. Izzetoglu K. Onaral B. Pourrezaei K. (2006). Functional near infrared spectroscopy: an emerging neuroimaging modality. IEEE Eng. Med. Biol. Mag. 25, 54–62 16898659 10.1109/memb.2006.1657788

[B15] Chance B. Zhuang Z. UnAh C. Alter C. Lipton L. (1993). Cognition-activated low-frequency modulation of light absorption in human brain. Proc. Natl. Acad. Sci. U.S.A. 90, 3770–3774 10.1073/pnas.90.8.3770 8475128 PMC46383

[B16] Christoff K. Gabrieli J. D. E. (2000). The frontopolar cortex and human cognition: Evidence for a rostrocaudal hierarchical organization within the human prefrontal cortex. Psychobiology 28, 168–186

[B17] Collette F. Van der Linden M. (2002). Brain imaging of the central executive component of working memory. Neurosci. Biobehav. Rev. 26, 105–125 10.1016/S0149-7634(01)00063-X 11856556

[B18] Collette F. Hogge M. Salmon E. Van der Linden M. (2006). Exploration of the neural substrates of executive functioning by functional neuroimaging. Neuroscience 139, 209–221 10.1016/j.neuroscience.2005.05.035 16324796

[B19] Contreras-Vidal J. L. Kerick S. E. (2004). Independent component analysis of dynamic brain responses during visuomotor adaptation. Neuroimage 21, 936–945 10.1016/j.neuroimage.2003.10.037 15006660

[B20] Coyle S. M. Ward T. E. Markham C. M. (2007). Brain-computer interface using a simplified functional near-infrared spectroscopy system. J. Neural Eng. 4, 219–226 10.1088/1741-2560/4/3/007 17873424

[B21] Coyle S. Ward T. E. Markham C. McDarby G. (2004). On the suitability of near-infrared (NIR) systems for next-generation brain-computer interfaces. Physiol. Meas. 25, 815–822 15382823 10.1088/0967-3334/25/4/003

[B22] Gentili R. J. Bradberry T. J. Hatfield B. D. Contreras-Vidal J. L. (2009). Brain biomarkers of motor adaptation using phase synchronization, in Proceedings of the 31st Annual International Conference of the IEEE Engineering in Medicine and Biology Society (EMBS'09), (Minneapolis, MN), 5930–5933 10.1109/IEMBS.2009.5334743 19965060

[B23] Gentili R. J. Bradberry T. J. Oh H. Hatfield B. D. Contreras-Vidal J. L. (2011). Cerebral cortical dynamics during visuomotor transformation: adaptation to a cognitive-motor executive challenge. Psychophysiology 48, 813–824 10.1111/j.1469-8986.2010.01143.x 20964696

[B24] Gentili R. J. Hadavi C. Ayaz H. Shewokis P. A. Contreras-Vidal J. L. (2010a). Hemodynamic correlates of visuomotor adaptation by functional near infrared spectroscopy, in Proceedings of the 32nd Annual International Conference of the IEEE Engineering in Medicine and Biology Society (EMBS'10), (Buenos Aires), 2918–2921 10.1109/IEMBS.2010.5626284 21095985

[B25] Gentili R. J. Oh H. Bradberry T. J. Hatfield B. D. Contreras-Vidal J. L. (2010b). Signal processing for non-invasive brain biomarkers of sensorimotor performance and brain monitoring, in Signal Processing, ed Miron S. (Vienna: In-Tech), 461–502

[B26] Georgopoulos A. P. Karageorgiou E. Leuthold A. C. Lewis S. M. Lynch J. K. Alonso A. A. (2007). Synchronous neural interactions assessed by magnetoencephalography: a functional biomarker for brain disorders. J. Neural Eng. 4, 349–355 10.1088/1741-2560/4/4/001 18057502

[B27] Ghahremani D. G. Monterosso J. Jentsch J. D. Bilder R. M. Poldrack R. A. (2010). Neural components underlying behavioral flexibility in human reversal learning. Cereb. Cortex 20, 1843–1852 10.1093/cercor/bhp247 19915091 PMC2901019

[B28] Ghilardi M. F. Ghez C. Dhawan V. Moeller J. Mentis M. Nakamura T. (2000). Patterns of regional brain activation associated with different forms of motor learning. Brain Res. 871, 127–145 10.1016/S0006-8993(00)02365-9 10882792

[B29] Graydon F. X. Friston K. J. Thomas C. G. Brooks V. B. Menon R. S. (2005). Learning-related fMRI activation associated with a rotational visuo-motor transformation. Cogn. Brain Res. 22, 373–383 15722208 10.1016/j.cogbrainres.2004.09.007

[B30] Hatakenaka M. Miyai I. Mihara M. Sakoda S. Kubota K. (2007). Frontal regions involved in learning of motor skill–a functional NIRS study. Neuroimage 34, 109–116 10.1016/j.neuroimage.2006.08.014 17067821

[B31] Hatfield B. D. Haufler A. J. Hung T. M. Spalding T. W. (2004). Electroencephalographic studies of skilled psychomotor performance. J. Clin. Neurophysiol. 21, 144–156 10.1097/00004691-200405000-00003 15375345

[B32] Hintze J. (2012). NCSS 8. Kaysville: NCSS, LLC

[B33] Huynh H. Feldt L. S. (1976). Estimation of the box correction for degrees of freedom from sample data in randomized block and split plot designs. J. Educ. Behav. Stat. 1, 69–82

[B34] Ikegami T. Taga G. (2008). Decrease in cortical activation during learning of a multi-joint discrete motor task. Exp. Brain Res. 191, 221–236 10.1007/s00221-008-1518-2 18679662

[B35] Izzetoglu M. Bunce S. C. Izzetoglu K. Onaral B. Pourrezaei K. (2007). Functional brain imaging using near-infrared technology. IEEE Eng. Med. Biol. Mag. 26, 8–46 17672230 10.1109/memb.2007.384094

[B36] Izzetoglu M. Chitrapu P. Bunce S. Onaral B. (2010). Motion artifact cancellation in NIR spectroscopy using discrete Kalman filtering. Biomed. Eng. Online 9:16 10.1186/1475-925X-9-16 20214809 PMC2846950

[B37] Izzetoglu S. M. Izzetoglu K. Bunce S. Ayaz H. Devaraj A. Onaral B. (2005). Functional near-infrared neuroimaging. IEEE Trans. Neural Syst. Rehabil. Eng. 13, 153–159 16003893 10.1109/TNSRE.2005.847377

[B38] James D. R. C. Leff D. R. Orihuela-Espina F. Kwok K. W. Mylonas G. P. Athanasiou T. (2012). Enhanced frontoparietal network architectures following ‘gaze-contingent’ versus ‘free-hand’ motor learning. Neuroimage 64, 267–276 10.1016/j.neuroimage.2012.08.056 22960153

[B39] James D. Orihuela-Espina F. Leff D. Mylonas G. Kwok K. W. Darzi A. (2010). Cognitive burden estimation for visuomotor learning with fNIR. Med. Image Comput. Comput. Assist. Interv. 13, 319–326 20879415 10.1007/978-3-642-15711-0_40

[B40] Kagerer F. A. Contreras-Vidal J. L. (2009). Adaptation of sound localization induced by rotated visual feedback in reaching movements. Exp. Brain Res. 193, 315–321 10.1007/s00221-008-1630-3 19048242 PMC3203351

[B41] Kluzik J. A. Diedrichsen J. Shadmehr R. Bastian A. J. (2008). Reach adaptation: what determines whether we learn an internal model of the tool or adapt the model of our arm? J. Neurophysiol. 100, 1455–1464 10.1152/jn.90334.2008 18596187 PMC2544452

[B42] Kluzik J. Diedrichsen J. Shadmehr R. Bastian A. J. (2008). Reach adaptation: what determines whether we learn an internal model of the tool or adapt the model of our arm? J. Neurophysiol. 100, 1455–1464 10.1152/jn.90334.2008 18596187 PMC2544452

[B43] Lacourse M. G. Orr E. L. Cramer S. C. Cohen M. J. (2005). Brain activation during execution and motor imagery of novel and skilled sequential hand movements. Neuroimage 27, 505–519 10.1016/j.neuroimage.2005.04.025 16046149

[B44] Leff D. R. Elwell C. E. Orihuela-Espina F. Atallah L. Delpy D. T. Darzi A. W. (2008a). Changes in prefrontal cortical behaviour depend upon familiarity on a bimanual co-ordination task: an fNIR study. Neuroimage 9, 805–813 10.1016/j.neuroimage.2007.09.032 17964187

[B45] Leff D. R. Orihuela-Espina F. Atallah L. Athanasiou T. Leong J. J. Darzi A. W. (2008b). Functional prefrontal reorganization accompanies learning-associated refinements in surgery: a manifold embedding approach. Comput. Aided Surg. 13, 325–339 10.3109/10929080802531482 18991082

[B46] Leff D. R. Orihuela-Espina F. Elwell C. E. Athanasiou T. Delpy D. T. Darzi A. W. (2011). Assessment of the cerebral cortex during motor task behaviours in adults: a systematic review of functional near infrared spectroscopy (fNIR) studies. Neuroimage 54, 2922–2936 10.1016/j.neuroimage.2010.10.058 21029781

[B47] Miller E. Cohen J. D. (2001). An integrative theory of prefrontal cortex function. Ann. Rev. Neurosci. 24, 167–202 10.1146/annurev.neuro.24.1.167 11283309

[B48] Miyai I. Yagura H. Hatakenaka M. Oda I. Konishi I. Kubota K. (2003). Longitudinal optical imaging study for locomotor recovery after stroke. Stroke 34, 2866–2870 10.1161/01.STR.0000100166.81077.8A 14615624

[B49] Miyake A. Friedman N. P. Emerson M. J. Witzki A. H. Howerter A. (2000). The unity and diversity of executive functions and their contribution to complex ‘frontal lobe’ tasks: a latent variable analysis. Cog. Psychol. 41, 49–100 10.1006/cogp.1999.0734 10945922

[B50] Morihiro M. Tsubone T. Wada Y. (2009). Relation between NIRS signal and motor capability, in Proceedings of the 31st Annual International Conference of the IEEE Engineering in Medicine and Biology Society (EMBS'09), (Minneapolis, MN), 3991–3994 10.1109/IEMBS.2009.5333526 19964088

[B51] Ohuchida K. Kenmotsu H. Yamamoto A. Sawada K. Hayami T. Morooka K. (2009). The frontal cortex is activated during learning of endoscopic procedures. Surg. Endosc. 23, 2296–2301 10.1007/s00464-008-0316-z 19172351

[B52] Perfetti B. Moisello C. Landsness E. C. Kvint S. Lanzafame S. Onofrj M. (2011). Modulation of gamma and theta spectral amplitude and phase synchronization is associated with the development of visuo-motor learning. J. Neurosci. 31, 14810–14819 10.1523/JNEUROSCI.1319-11.2011 21994398 PMC3206224

[B53] Pfurtscheller G. Stancák A. Neuper C. (1996). Event-related synchronization (ERS) in the alpha band–an electrophysiological correlate of cortical idling: a review. Int. J. Psychophysiol. 24, 39–46 10.1016/S0167-8760(96)00066-9 8978434

[B54] Power S. D. Kushki A. Chau T. (2012). Automatic single-trial discrimination of mental arithmetic, mental singing and the no-control state from prefrontal activity: toward a three-state NIRS-BCI. BMC Res. Notes 5:141 10.1186/1756-0500-5-141 22414111 PMC3359174

[B55] Rietschel J. C. (2011). Psychophysiological Investigation of Attentional Processes During Motor Learning. Ph.D. dissertation, University of Maryland. http://hdl.handle.net/1903/11906

[B56] Roche-Labarbe N. Wallois F. Ponchel E. Kongolo G. Grebe R. (2007). Coupled oxygenation oscillation measured by NIRS and intermittent cerebral activation on EEG in premature infants. Neuroimage 36, 718–727 10.1016/j.neuroimage.2007.04.002 17482837

[B57] Seidler R. D. Noll D. C. (2008). Neuroanatomical correlates of motor acquisition and motor transfer. J. Neurophysiol. 99, 1836–1845 10.1152/jn.01187.2007 18272874

[B58] Seidler R. D. Noll D. C. Chintalapati P. (2006). Bilateral basal ganglia activation associated with sensorimotor adaptation. Exp. Brain Res. 175, 544–555 10.1007/s00221-006-0571-y 16794848

[B59] Shadmehr R. Holcomb H. H. (1997). Neural correlates of motor memory consolidation. Science 277, 821–825 10.1126/science.277.5327.821 9242612

[B60] Shadmehr R. Holcomb H. H. (1999). Inhibitory control of competing motor memories. Exp. Brain Res. 126, 235–251 10.1007/s002210050733 10369146

[B61] Shimamura A. P. (2000). The role of prefrontal cortex in dynamic filtering. Psychobiology 28, 207–218

[B62] Sitaram R. Zhang H. Guan C. Thulasidas M. Hoshi Y. Ishikawa A. (2007). Temporal classification of multichannel near-infrared spectroscopy signals of motor imagery for developing a brain-computer interface. Neuroimage 34, 1416–1427 10.1016/j.neuroimage.2006.11.005 17196832

[B63] Strangman G. Boas D. A. Sutton J. P. (2002). Non-invasive neuroimaging using near-infrared light. Biol. Psychiatry 52, 679–693 12372658 10.1016/s0006-3223(02)01550-0

[B64] Suzuki M. Miyai I. Ono T. Oda I. Konishi I. Kochiyama T. (2004). Prefrontal and premotor cortices are involved in adapting walking and running speed on the treadmill: an optical imaging study. Neuroimage 23, 1020–1026 10.1016/j.neuroimage.2004.07.002 15528102

[B65] Sweeney K. Ayaz H. Ward T. Izzetoglu M. McLoone S. Onaral B. (2012). A methodology for validating artifact removal techniques for physiological signals. IEEE Trans. Inf. Technol. Biomed. 16, 918–926 10.1109/TITB.2012.2207400 22801522

[B66] Takeda K. Gomi Y. Imai I. Shimoda N. Hiwatari M. Kato H. (2007). Shift of motor activation areas during recovery from hemiparesis after cerebral infarction: a longitudinal study with near-infrared spectroscopy. Neurosci. Res. 59, 136–144 10.1016/j.neures.2007.06.1466 17681629

[B67a] Umeyama S. Yamada T. (2013). Detection of an unstable and/or a weak probe contact in a multichannel functional near-infrared spectroscopy measurement. J. Biomed. Opt. 18:047003 10.1117/1.JBO.18.4.047003 23552638

[B67] Villringer A. Chance B. (1997). Non-invasive optical spectroscopy and imaging of human brain function. Trends Neurosci. 20, 435–442 10.1016/S0166-2236(97)01132-6 9347608

[B68] Zhang Q. Strangman G. E. Ganis G. (2009). Adaptive filtering to reduce global interference in non-invasive NIRS measures of brain activation: how well and when does it work? Neuroimage 45, 788–794 10.1016/j.neuroimage.2008.12.048 19166945 PMC2671198

